# Mannitol reduces nephron loss after warm renal ischemia in a porcine model

**DOI:** 10.1186/s12894-018-0328-5

**Published:** 2018-03-06

**Authors:** José A. Damasceno-Ferreira, Leonardo A. S. Abreu, Gustavo R. Bechara, Waldemar S. Costa, Marco A. Pereira-Sampaio, Francisco J. B. Sampaio, Diogo B. De Souza

**Affiliations:** 1 Urogenital Research Unit, Rio de Janeiro State University, Rio de Janeiro, RJ Brazil; 2 Department of Veterinary Clinical Pathology, Fluminense Federal University, Niterói, RJ Brazil; 3 Faculty of Medicine, Estacio de Sá University, Rio de Janeiro, RJ Brazil; 4 Department of Morphology, Fluminense Federal University, Niteroi, RJ Brazil

**Keywords:** Kidney, Mannitol, Partial nephrectomy, Warm ischemia, Swine

## Abstract

**Background:**

Mannitol has been employed to ameliorate renal warm ischemia damage during partial nephrectomy, however, there is limited scientific evidence to support the use of mannitol during partial nephrectomy. The objective of the present study was to investigate the glomerular number after renal warm ischemia, with and without the use of mannitol in a Pig Model.

**Methods:**

Twenty-four male pigs were assigned into three groups. Eight animals were allocated to the sham group that was subjected to laparoscopic dissection of the left renal hilum, without renal ischemia. Eight animals were allocated to the ischemia group that had the left renal hilum clamped for 30 min through laparoscopic access. Eight animals received mannitol (250 mg/kg) before the occlusion of renal hilum for 30 min. The kidneys were collected after the euthanasia of the pigs 21 days post surgery. The right kidney was utilized as a self-control for each animal. Serum creatinine, urea levels, the weight and volume of the kidneys were measured. Glomerular volumetric density, volume-weighted glomerular volume, and cortical volume were quantified through stereological methods and employed to determine the number of nephrons per kidney. Student’s *t* test and ANOVA were used for statistical analysis.

**Results:**

In the ischemia group, the left kidney recorded a reduction of 24.6% (290, 000 glomeruli) in the number of glomeruli in comparison to the right kidney. Kidneys subjected to ischemia also displayed decreased weight and volume in comparison to the sham and mannitol groups. No difference was observed between the left and right kidneys from the sham and mannitol groups. Further, no distinction in serum creatinine and urea among the groups was observed.

**Conclusion:**

The use of mannitol significantly reduces nephron loss during warm ischemia in pigs.

## Background

Despite the development of new techniques for minimally invasive partial nephrectomy, renal warm ischemia is often necessary to obtain an adequate operative field [1]. However, renal ischemia during partial nephrectomy is associated with post-operative functional decline [2].

A maximum duration of 25 min for warm ischemia has been proposed for preventing renal damage [3]. Furthermore, recent studies have shown that the quality and quantity of remnant renal parenchyma is of great importance to predict renal function [4]. Thus, various methods have been employed to prevent damage to the remnant kidney parenchyma after prolonged warm renal ischemia [5–8].

Mannitol has been employed to ameliorate the renal damage caused by warm ischemia during partial nephrectomies. Although there is limited scientific evidence to validate the application of mannitol to preserve kidney function during partial nephrectomy, almost 80% of groups that perform partial nephrectomy routinely apply mannitol as an ameliorating agent [9]. According to some investigations, renal function exhibits no difference in relation to mannitol administration during renal ischemia in partial nephrectomy [10, 11]. However, there is a lack of quantitative morphological studies exploring the effects of mannitol to ameliorate damage caused during renal warm ischemia. For this experiment, swine was employed as an animal model, since it is considered the most adequate model for comparison with human kidney’s anatomy and physiology [12, 13] Thus, the aim of this study was to investigate the number of glomeruli, applying an unbiased stereological method, post renal warm ischemia with and without the administration of mannitol, in a porcine model.

## Methods

Twenty-four male domestic pigs weighing 25 kg were included in this study. All experiments were performed in adherence to the Brazilian law for scientific use of animals, and this project was formally approved by the local Ethics Committee for animal experimentation (CEUA-048-2011). Animals were accommodated in groups of six in appropriate facilities, with air conditioning, food, and water ad libitum.

The animals were randomly assigned into three experimental groups of eight animals each. Group sham (S) was subjected to kidney and hilar dissection but not renal ischemia. Group ischemia (I) was subjected to 30 min of renal warm ischemia. Group mannitol (M) was also subjected to 30 min of renal warm ischemia, but mannitol (250 mg/kg, IV) [14] was administrated 15 min before the pedicle clamping.

The left kidney was accessed laparoscopicaly with a transperitoneal approach under general anesthesia and aseptic technique, [12]. Renal vessels were clamped *en bloc* in groups I and M with a laparoscopic Satinsky clamp. After 30 min of ischemia, the vascular clamp was removed, and the normal color of the kidney was verified through observation. In the sham group, all steps (except the hilar clamping) were performed; subsequent to the dissection of the renal pedicle, the animals were maintained under anesthesia for 30 min without renal ischemia. The right kidneys were not manipulated during the experiment and were served as controls. The animals were administered a single dose of penicillin benzathine (Benzetacil, Eurofarma, São Paulo, Brazil) at 400, 000 UI/Kg subsequent to anesthetic induction and tramadol hydrochloride (Tramal, Pfizer, Guarulhos, Brazil) at 4 mg/Kg twice a day for 48 h post surgery. Food and water were offered ad libitum six hours after the procedure. The recovery to normal ambulation required up to four hours after the surgery. Serum creatinine and urea levels were determined before surgery and on post-operative days 10 and 21 to assess renal function. For this purpose, animals were restrained and blood was collected through venipuncture. Serum was separated through the technique of centrifugation and stored at −20^o^ C until analysis.

The animals were evaluated on a daily basis for 21 days after surgery, and subsequent to this period, were euthanized through anesthetic overdose (sodium thiopental 200 mg/kg IV). The kidneys were harvested, weighed, and their volumes were measured with the Scherle’s method [15]; subsequently, the organs were fixed by immersion in 4% buffered formaldehyde for stereological analyses. All histological analyses were performed by a blinded observer. Samples were randomly collected from the cortical region of these 48 kidneys and were processed through routine histological methods. The specimens were paraffin-embedded, sectioned at 5-μm thickness, and stained with hematoxylin and eosin. The cortical-medullar ratio was estimated employing the point-counting-method according to the Cavalieri principle [16]. The absolute cortical volume (CV) was achieved through the product of the cortical-medullar ratio and renal volume.

From each kidney, 25 histological fields obtained from different sections of the renal cortex were photographed with a digital camera (DP70, Olympus, Tokyo, Japan) coupled to a microscope (BX51, Olympus). Glomerular volumetric density (Vv [glom]) was estimated by the point-counting technique with a M42 test-system [17, 18].

The volume-weighted mean glomerular volume (VWGV) was estimated using the point-sampled intercept method [16, 17, 19], analyzing 50 glomeruli per kidney.

The estimation of the total number of glomeruli per kidney was achieved through the product of CV and Vv [glom] and the division of the quotient by the VWGV [16, 19].

For each stereological parameter, left kidneys were compared with the right organs of each group with the Student’s *t* test. Mean creatinine and urea serum levels were compared between groups by employing one-way ANOVA and between different experimental instances (0, 10, and 21 days respectively) through repeated measures ANOVA. For all comparisons, *p* < 0.05 was considered significant. Data were expressed as mean ± standard deviation. Analyses were performed using GraphPad Prism 5.0 (GraphPad Software, San Diego, USA).

## Results

All animals recovered effectively from the surgeries and were included for the evaluation of allanalyzed parameters. No adverse events were observed. No variations in serum creatinine and urea levels were observed among the studied groups (Table 1).

**Table 1:** Serum creatinine levels of pigs subjected to sham surgery or to renal ischemia with or without mannitol administration

Creatinine		Preoperative	10 days Post-operative	21 days Post-operative	*p* value
	Sham	1.52 ± 0.4	1.10 ± 0.1	1.39 ± 0.7	0.62
	Ischemia	1.13 ± 0.3	1.13 ± 0.2	1.20 ± 0.3	0.85
	Mannitol	1.3 ± 0.5	0.92 ± 0.2	0.91 ± 0.1	0.10
	*p* value	0.31	0.16	0.16	
		Preoperative	10 days Post-operative	21 days Post-operative	*p* value
Urea	Sham	37.0 ± 1.4	38.2 ± 8.2	41.0 ± 6.8	0.63
	Ischemia	41.9 ± 8.2	41.2 ± 7.2	42 ± 7.2	0.98
	Mannitol	33.4 ± 4.9	35.7 ± 9.0	35.9 ± 7.0	0.82
	*p* value	0.09	0.45	0.21	

Data expressed as mean ± S.D.

The weight and volume of the left kidney for group I reduced by 6.2% and 6.3% respectively, in comparison to the right kidney. For group S as well as group M, no difference was observed between the weight and volume of the kidneys.

The cortical-medullar ratio and absolute CV were the only factors that recorded a difference among left and right kidneys of group I, with the left kidney displaying a 2.3% and 8.3% decrease in these parameters respectively. For the other groups, no difference was noted regarding these parameters. Regarding Vv [glom] and VWGV, no difference was found across all groups.

**Fig. 1:**
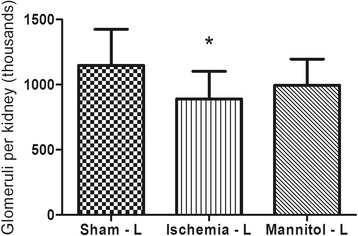
Number of glomeruli in the left kidneys of pigs subjected to sham surgery or warm ischemia for 30 min with and without mannitol pre-treatment. *p < 0.05 in comparison to contra lateral control. Data are expressed as mean (boxes) ± standard deviation (error bars)

Finally, the total number of glomeruli in left kidneys of group I, a 24.6% decrease in comparison to the right kidneys was observed. This represented a loss of approximately 290, 000 glomeruli caused by warm ischemia for 30 min (Fig. 1). However, in the group subjected to the same duration of ischemia exposure, but received mannitol pre-treatment, no difference was observed, along with group S. All stereological data are listed in Table 2.

**Table 2:** Stereological data of right and left kidneys of pigs subjected to sham surgery or to left renal ischemia with or without mannitol administration

	Sham			Ischemia			Mannitol		
	Right	Left	*p*-value	Right	Left	*p*-value	Right	Left	*p*-value
Kidney weight (g)	56.8 ± 4.9	58.2 ± 8.5	0.52	59.2 ± 10.9	55.5 ± 11.0	0.008	58.5 ± 10.4	55.4 ± 8.5	0.06
Kidney volume (ml)	54.4 ± 4.1	55.2 ± 7.7	0.70	56.6 ± 9.8	53.0 ± 10.5	0.007	55.4 ± 9.8	52.5 ± 8.1	0.09
Cortical-medullar ratio (%)	71.6 ± 2.3	70.4 ± 4.1	0.32	71.8 ± 2.4	70.1 ± 2.2	0.04	71.4 ± 4.0	71.9 ± 3.3	0.72
Cortical volume (ml)	38.9 ± 3.3	38.9 ± 6.5	0.98	40.6 ± 7.4	37.2 ± 7.9	0.002	39.3 ± 5.9	37.8 ± 6.2	0.17
Vv [glom] (%)	3.79 ± 0.5	3.72 ± 0.5	0.68	3.59 ± 0.4	3.08 ± 0.9	0.16	4.06 ± 0.9	3.72 ± 0.8	0.37
VWGV (10^5^ μm^3^)	13.4 ± 1.6	12.7 ± 1.1	0.25	12.5 ± 2.4	12.4 ± 1.2	0.94	14.1 ± 3.9	14.3 ± 3.9	0.86
Glomeruli (millions)	1.10 ± 0.1	1.14 ± 0.2	0.68	1.18 ± 0.2	0.89 ± 0.2	0.04	1.18 ± 0.4	0.99 ± 0.2	0.18

Data expressed as mean ± S.D.

## Discussion

Warm ischemia was identified as the “ultimate enemy” for partial nephrectomy [20], and several methods to ameliorate its negative aspects have been proposed [5–8]. Although mannitol is largely employed for this purpose [9], its effects for ischemia protection during partial nephrectomy were only recently investigated [10, 11]. This is the first study that shows the beneficial effects of mannitol for preserving the renal functional units post warm ischemia.

Both previous studies on this issue indicated that the use of mannitol during partial nephrectomies does not affect clinically significant improvements in renal function preservation [10, 11]. They were retrospective studies that presented the results of estimated glomerular filtration rate of patients subjected to partial nephrectomy with or without mannitol administration. Therefore, their negative results may be due to the heterogeneity of the patients and laboratory analysis. Although the animal model presents certain limitations, the present study was randomly conducted with several appropriate control factors that may affect the results, such as age, weight, and nutritional status, warm ischemia time, mannitol dosage, and performed analysis.

Furthermore, in the present study, we used unbiased stereological methods to determine the number of glomeruli on each kidney that could be considered equal to the number of nephrons [16, 19]. As a “nephron-sparing surgery,” the main objective of partial nephrectomy is the treatment of the renal tumor while sparing as most nephrons as possible. In accordance with this objective, one of the most adequate measurements to study the impact of warm ischemia for partial nephrectomy would be the determination of the number of nephrons [17].

The preservation of renal nephrons following warm ischemia by mannitol usage may rely on different mechanisms. Mannitol is most recognized as an osmotic diuretic, and diuretics (not only mannitol but also furosemide) are known to inhibit tubular reabsorption and decrease parenchymal oxygen demand [21]. Thus, different diuretics have been employed before renal warm ischemia [9]. Moreover, mannitol is an intravascular volume expander, associated with the increased renal blood flow [22]. As more blood flows into the kidney, more oxygen is delivered to renal cells, and this is considered beneficial for an organ supposed to be subjected to ischemia. Finally, mannitol is also considered an antioxidant, capable of scavenging the hydroxyl radical, thus reducing oxidant-derived injury in several organs [23, 24]. As reactive oxygen species are largely produced during renal warm ischemia, the group that received mannitol in the present study may experience non-significant reduction in the number of nephrons due to the antioxidant properties of mannitol.

Despite the potential advantages of the use of mannitol during warm ischemia for partial nephrectomy displayed in the present study, some issues should be addressed. As stated by Omae et al., [10] omitting the use of mannitol offers some advantages, including reduction of operative time and procedural costs. Further, some complications with mannitol administration during partial nephrectomy have been reported [25]. Thus, we should note that mannitol usage is not free of charge. However, we should emphasize that mannitol infusion can be planned during surgery to reduce operative time, and also, the cost of mannitol is minimal, especially when compared to the overall costs of laparoscopic partial nephrectomy.

The results of our study support the application of mannitol as a renal warm ischemia protective agent to be employed during partial nephrectomy. Regardless, further evidence confirming or refuting these results is required. The effects of mannitol should be studied in other experimental situations such as single kidney models, renal insufficiency, and selective clamping techniques. Further, this is an animal study, and its results should not be directly transposed to humans. Although the swine constitutes the most adequate model for comparison with human kidney’s anatomy and physiology [12, 13], is the fact remains that this is study was conducted in an experimental setting and different from clinical practice.

## Conclusion

In conclusion, we discovered that warm ischemia of 30 min in a Pig Model determined a loss of nearly one quarter of renal nephrons. However, the application of mannitol prevented significant nephron loss during warm ischemia.

## Abbreviations

CV: Absolute cortical volume
I: Group ischemia
M: Group mannitol
S: Group sham
Vv [glom]: Glomerular volumetric density
VWGV: Volume-weighted mean glomerular volume

## References

[1] Haber GP, Gill IS (2006). Laparoscopic partial nephrectomy: contemporary technique and outcomes. Eur Urol.

[2] Mir MC, Ercole C, Takagi T, Zhang Z, Velet L, Remer EM, Demirjian S, Campbell SC (2015). Decline in renal function after partial nephrectomy: etiology and prevention. J Urol.

[3] Rod X, Peyronnet B, Seisen T, Pradere B, Gomez FD, Verhoest G, Vaessen C, De La Taille A, Bensalah K, Roupret M. Impact of ischaemia time on renal function after partial nephrectomy: a systematic review. BJU Int. 2016; 10.1111/bju.13580 27409986

[4] Thompson RH, Lane BR, Lohse CM, Leibovich BC, Fergany A, Frank I, Gill IS, Blute ML, Campbell SC (2012). Renal function after partial nephrectomy: effect of warm ischemia relative to quantity and quality of preserved kidney. Urol.

[5] Cohen J, Dorai T, Ding C, Batinic-Haberle I, Grasso M (2013). The administration of renoprotective agents extends warm ischemia in a rat model. J Endourol.

[6] Gill IS, Patil MB, Abreu AL, Ng C, Cai J, Berger A, Eisenberg MS, Nakamoto M, Ukimura O, Goh AC (2012). Zero ischemia anatomical partial nephrectomy: a novel approach. J Urol.

[7] Keel CE, Wang Z, Colli J, Grossman L, Majid D, Lee BR. Protective effects of reducing renal ischemia-reperfusion injury during renal hilar clamping: use of allopurinol as a nephroprotective agent. *Urol* 2013, **81**. 210:e215–0. 10.1016/j.urology.2012.08.016 23153953

[8] Wang Z, Colli JL, Keel C, Bailey K, Grossman L, Majid D, Lee BR (2012). Isoprostane: quantitation of renal ischemia and reperfusion injury after renal artery clamping in an animal model. J Endourol.

[9] Cosentino M, Breda A, Sanguedolce F, Landman J, Stolzenburg JU, Verze P, Rassweiler J, Van Poppel H, Klingler HC, Janetschek G (2013). The use of mannitol in partial and live donor nephrectomy: an international survey. W J urol.

[10] Omae K, Kondo T, Takagi T, Iizuka J, Kobayashi H, Hashimoto Y, Tanabe K (2014). Mannitol has no impact on renal function after open partial nephrectomy in solitary kidneys. Int J Urol.

[11] Power NE, Maschino AC, Savage C, Silberstein JL, Thorner D, Tarin T, Wong A, Touijer KA, Russo P, Coleman JA (2012). Intraoperative mannitol use does not improve long-term renal function outcomes after minimally invasive partial nephrectomy. Urol.

[12] de Souza DB, Abilio EJ, Costa WS, Sampaio MA, Sampaio FJ (2011). Kidney healing after laparoscopic partial nephrectomy without collecting system closure in pigs. Urol.

[13] Pereira-Sampaio MA, Favorito LA, Sampaio FJ (2004). Pig kidney: anatomical relationships between the intrarenal arteries and the kidney collecting system. Applied study for urological research and surgical training. J Urol.

[14] Khoury W, Namnesnikov M, Fedorov D, Abu-Gazala S, Weinbroum AA (2010). Mannitol attenuates kidney damage induced by xanthine oxidase-associated pancreas ischemia-reperfusion. J Surg Res.

[15] Ribeiro CT, Milhomem R, De Souza DB, Costa WS, Sampaio FJ, Pereira-Sampaio MA (2014). Effect of antioxidants on outcome of testicular torsion in rats of different ages. J Urol.

[16] Souza DB, Costa WS, Cardoso LE, Benchimol M, Pereira-Sampaio MA, Sampaio FJ (2013). Does prolonged pneumoperitoneum affect the kidney? Oxidative stress, stereological and electron microscopy study in a rat model. Int Braz J Urol.

[17] de Souza DB, de Oliveira LL, da Cruz MC, Abilio EJ, Costa WS, Pereira-Sampaio MA, Sampaio FJ (2012). Laparoscopic partial nephrectomy under warm ischemia reduces the glomerular density in a pig model. J Endourol.

[18] de Souza DB, Silva D, Cortez CM, Costa WS, Sampaio FJ (2012). Effects of chronic stress on penile corpus cavernosum of rats. J Androl.

[19] Benchimol de Souza D, Silva D, Marinho Costa Silva C, Barcellos Sampaio FJ, Silva Costa W, Martins Cortez C (2011). Effects of immobilization stress on kidneys of Wistar male rats: a morphometrical and stereological analysis. Kidney & blood pressure research.

[20] Pignot G, Bouliere F, Patard JJ (2010). Warm ischaemia: the ultimate enemy for partial nephrectomy. Eur Urol.

[21] Gelman S (1996). Does mannitol save the kidney. Anesth Analg.

[22] Zager RA, Mahan J, Merola AJ (1985). Effects of mannitol on the postischemic kidney. Biochemical, functional, and morphologic assessments. Laboratory investigation; a journal of technical methods and pathology.

[23] England MD, Cavarocchi NC, O'Brien JF, Solis E, Pluth JR, Orszulak TA, Kaye MP, Schaff HV (1986). Influence of antioxidants (mannitol and allopurinol) on oxygen free radical generation during and after cardiopulmonary bypass. Circulation.

[24] Haraldsson G, Sorensen V, Nilsson U, Pettersson S, Rashid M, Schersten T, Akerlund S, Jonsson O (1995). Effect of pre-treatment with desferrioxamine and mannitol on radical production and kidney function after ischaemia-reperfusion. A study on rabbit kidneys. Acta Physiol Scand.

[25] Erickson BA, Yap RL, Pazona JF, Hartigan BJ, Smith ND (2007). Mannitol extravasation during partial nephrectomy leading to forearm compartment syndrome. Int Braz J Urol.

